# Zonisamide can ameliorate the voltage-dependence alteration of the T-type calcium channel Ca_V_3.1 caused by a mutation responsible for spinocerebellar ataxia

**DOI:** 10.1186/s13041-020-00700-7

**Published:** 2020-11-26

**Authors:** Naoyuki Hara, Hiroyuki Morino, Yukiko Matsuda, Kenichi Satoh, Kouichi Hashimoto, Hirofumi Maruyama, Hideshi Kawakami

**Affiliations:** 1 Department of Clinical Neuroscience and Therapeutics, Graduate School of Biomedical and Health Sciences, Hiroshima University, 1-2-3 Kasumi, Minami-ku, Hiroshima, Hiroshima 734-8551 Japan; 2 Department of Epidemiology, Research Institute for Radiation Biology and Medicine, Hiroshima University, 1-2-3 Kasumi, Minami-ku, Hiroshima, Hiroshima 734-8553 Japan; 3 The Center for Data Science Education and Research, Shiga University, 1-1-1 Banba, Hikone, Shiga 522-8522 Japan; 4 Department of Neurophysiology, Graduate School of Biomedical and Health Sciences, Hiroshima University, 1-2-3 Kasumi, Minami-ku, Hiroshima, Hiroshima 734-8551 Japan

**Keywords:** Spinocerebellar ataxia, SCA42, *CACNA1G*, T-type calcium channel, Ca_V_3.1, Tremor, Zonisamide

## Abstract

Spinocerebellar ataxia (SCA) 42 is caused by a mutation in *CACNA1G*, which encodes the low voltage-gated calcium channel Ca_V_3.1 (T-type). Patients with SCA42 exhibit a pure form of cerebellar ataxia. We encountered a patient with the p.Arg1715His mutation, suffering from intractable resting tremor, particularly head tremor. This symptom improved with the administration of low-dose of zonisamide (ZNS), a T-type calcium channel blocker effective for treating Parkinson’s disease and epilepsy. Previous electrophysiological studies showed that the voltage dependence of this mutant Ca_V_3.1 was shifted toward the positive potential. This abnormal shift was considered a factor related to disease onset and symptoms. In this study, we performed whole-cell recordings of GFP-expressing HEK293T cells that expressed wild-type or mutant Ca_V_3.1 and investigated the changes in the abnormal shift of voltage dependence of the mutant Ca_V_3.1. The results showed that ZNS in an amount equivalent to the patient’s internal dose significantly ameliorated the abnormal shift in the mutant Ca_V_3.1, giving values close to those in the wild-type. On the other hand, ZNS did not affect the voltage dependence of wild-type Ca_V_3.1. Because Ca_V_3.1 is known to be involved in tremogenesis, modulation of the voltage dependence of mutant Ca_V_3.1 by ZNS might have contributed to improvement in the intractable tremor of our patient with SCA42. Moreover, efonidipine, another T-type calcium channel blocker, had no effect on tremors in our patient with SCA42 and did not improve the abnormal shift in the voltage dependence of the mutant Ca_V_3.1. This indicates that ZNS is distinct from other T-type calcium channel blockers in terms of modulation of the voltage dependence of the mutant Ca_V_3.1.

## Introduction

A recurrent mutation of p.Arg1715His in *CACNA1G*, which encodes the low voltage-gated calcium channel Ca_V_3.1 (T-type), alters the physiological properties of the channel and causes spinocerebellar ataxia 42 (SCA) [1–5]. This mutation is located in the S4 voltage-sensor segment of Ca_V_3.1. Patients with SCA42 present with a pure form of cerebellar ataxia, and some patients present with other symptoms such as dementia, truncal myoclonus, myokymia, and tremor [1–3].

We surveyed a family with SCA42, in which two patients presented with intractable resting tremors. We previously reported that a low-dose of zonisamide (ZNS) (25 mg/day p.o.) effectively suppressed intractable resting tremors, particularly head tremors, in one of the patients with SCA42 [6]. ZNS is used as an antiepileptic drug and for treating Parkinson’s disease (PD) in Japan [7]. ZNS is known to have various effects, including impacts on T-type voltage-dependent calcium channel (VDCC) activity [8]. Some previous electrophysiological studies suggested that ZNS reduces the Ca^2+^ current in a concentration-dependent manner and affects the voltage dependence of channel activity [9, 10]. Furthermore, Ca_V_3.1 is highly expressed in the inferior olive and cerebellum and is involved in the generation of tremor-related rhythms [11]. This suggests that the drug effects of ZNS are caused by the modulation of Ca_V_3.1 activity.

We previously reported that the mutation of SCA42 in *CACNA1G* shifted the voltage dependence of Ca_V_3.1 activation and inactivation to more depolarized potentials [1, 2]. Therefore, we predicted that ZNS could ameliorate this abnormal voltage dependence of Ca_V_3.1. To investigate this possibility, we transfected wild-type and mutant *CACNA1G* into HEK293T cells and recorded the current using the whole-cell patch-clamp technique. We found that ZNS shifted the voltage dependence of Ca_V_3.1 activation to a hyperpolarized potential. We also treated the cells with efonidipine, another T-type VDCC blocker that does not affect tremor suppression, and found that efonidipine did not affect the voltage dependence of Ca_V_3.1. These results suggest that ZNS is distinct from other T-type calcium channel blockers in terms of its modulation of the voltage dependence of the mutant Ca_V_3.1.

## Methods

### Expression vector

Wild-type CACNA1G (short isoform; BC110995.1, NM_198382.2) in the pCMV-SPORT6 plasmid (pCMV-SPORT6-CACNA1G) was purchased from Dharmacon (Lafayette, CO, USA). The mutation c.5075G>A, corresponding to c.5144G>A in the longest isoform (NM_018896.4), was introduced by site-directed mutagenesis using QuikChange Lightning (Agilent Technologies, Santa Clara, CA, USA) and verified by bidirectional sequencing. The IRES-EGFP sequence was amplified by PCR from the pIRES-EGFP plasmid and inserted after the termination codon in the cDNA sequence in pCMV-SPORT6-CACNA1G (pCMV-SPORT6-CACNA1G-IG) using an In-Fusion HD Cloning Kit (Takara Bio, Shiga, Japan).

### Cell culture, transformation, and immunofluorescence staining

HEK293T cells were maintained in Dulbecco’s modified Eagle’s medium (Nacalai Tesque, Kyoto, Japan) supplemented with 10% fetal bovine serum and penicillin/streptomycin at 37 °C in an incubator with 5% CO_2_. Cells for whole-cell patch clamping were grown in glass-bottom plates (μ-Dish 35 mm low; Ibidi, Martinsried, Germany) for 24 h following transfection with pCMV-SPORT6-CACNA1G-IG using Lipofectamine LTX (Thermo Fisher Scientific, Waltham, MA, USA).

### Electrophysiology

Whole-cell recordings were obtained from GFP-expressing HEK293T cells using an upright microscope (BX51WI; Olympus, Tokyo, Japan) equipped with an IR-CCD camera system (IR-1000; DAGE-MTI, Michigan, IN, USA) at room temperature. To confirm the reproducibility of our previous study results [1], we made whole-cell recordings from cells (approximately 20% of cells used in the experiment) without blinding. For the remaining experiments with added reagent, whole-cell recordings were performed with blinding. The intracellular solution was composed of 110 mM CsCl, 20 mM TEA-Cl, 5 mM EGTA, 10 mM HEPES, 4 mM MgCl_2_, 4 mM 2Na-ATP, and 0.4 mM 2Na-GTP (pH 7.3, adjusted with CsOH). The pipette access resistance was approximately 2–3 MΩ. To separate the Ca^2+^ current, HEK293T cells were bathed with an extracellular solution containing 4-AP and a high concentration of TEA-Cl, which are broad-spectrum and non-selective potassium channel blockers. The composition of the extracellular solution (control) used for voltage-dependent Ca^2+^ current recording was 10 mM NaCl, 105 mM TEA-Cl, 10 mM 4-AP, 2.5 mM KCl, 2 mM CaCl_2_, 1 mM MgSO_4_, 1.25 mM NaH_2_PO_4_, 26 mM NaHCO_3_, and 20 mM glucose; this solution was bubbled with 95% O_2_ and 5% CO_2_. Ionic currents were recorded with an EPC-10 (HEKA Elektronik, Lambrecht, Germany). T-type calcium channel currents were activated by stepwise depolarization after hyperpolarization to − 100 mV (300 ms). The signals were filtered at 3 kHz and digitized at 20 kHz. Online data acquisition and offline data analysis were performed using the PATCHMASTER software (HEKA Elektronik). The relative conductance and steady-state inactivation potentials were fitted by the following Boltzmann equations:

$$\frac{G}{{G}_{max}}=\frac{1}{1+exp\left(\left({V}_{half}-{V}_{m}\right)/k\right)}$$

$$\frac{I}{{I}_{max}}=\frac{1}{1+exp\left(\left({V}_{m}-{V}_{half}\right)/k\right)}$$

where *G* and *I* are the conductance and the current at an individual membrane potential, respectively, and *G*_*max*_ and *I*_*max*_ are the maximum conductance and the peak current, respectively. *V*_*half*_, *k*, and *V*_*m*_ are the half-conductance potential, slope factor, and membrane potential, respectively. The reversal potential of the Ca_V_3.1 current was estimated from the fitted lines of the peak amplitudes recorded at − 10, 0, + 10, + 20, and + 30 mV.

### Reagent addition

The effect of zonisamide sodium salt (Sigma-Aldrich, St. Louis, MO, USA) or efonidipine hydrochloride (Cosmo Bio, Tokyo, Japan) against the electrophysiological properties of mutant Ca_V_3.1 was examined by bathing the extracellular solution containing the reagent. The concentrations of ZNS were 0, 10, and 50 µM. This range of concentrations approximated the blood concentration of ZNS following administration of our patient, which was based on a report that the blood concentration and brain concentration are approximately equivalent [12]. The concentration of efonidipine was 5 µM, which was based on the plasma concentration in healthy adults orally administered the same amount of efonidipine as our patients and as described on the product sheet. To avoid the run-down and negative influence of insufficient washout of calcium channel blockers when recording the next cell, we obtained whole-cell recordings from cells without calcium channel blockers. Following the control experiment, we conducted whole-cell recordings from cells with calcium channel blockers. Once the reagent was applied, the dish was discarded, and the next experiment was conducted using a new dish. There was no significant difference in cell capacitance between groups.

### Statistical analyses

Multiple linear regression analysis was used to study the linear relationship between a dependent variable (Ca^2+^ current density, the half-conductance potential, or the slope factor) and two independent variables (the presence of mutation and the concentration of the reagent [ZNS or efonidipine]). The analyses were performed for the whole-cell recordings experiments using R version 3.6.0. Numerical data are indicated as the mean ± standard deviation (SD) or as the mean ± standard error of the mean (S.E.M). The level of significance was set at p < 0.05.

## Results

### ZNS ameliorated the electrophysiological properties of the mutant Ca_V_3.1

**Fig. 1:**
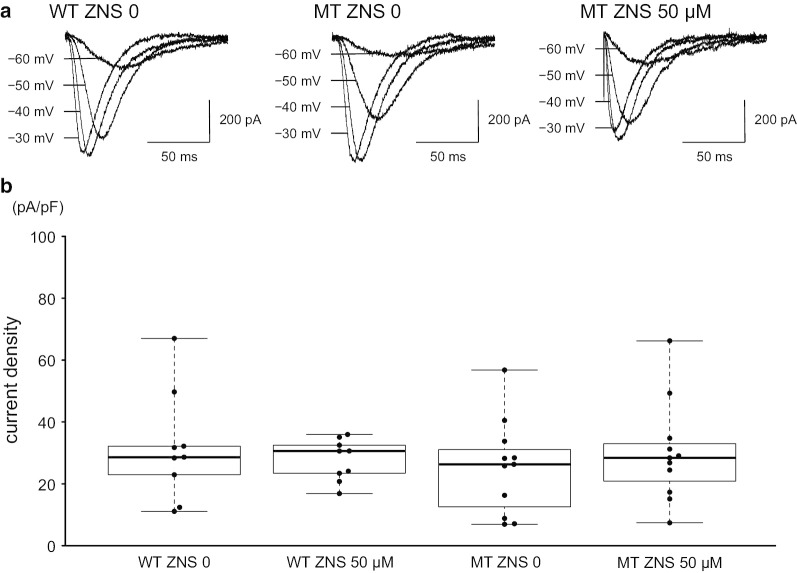
Effect of ZNS on mutant Ca_V_3.1. **a** Representative traces of T-type VDCC currents recorded from HEK293T cells expressing WT without ZNS (left), MT without ZNS (middle), or MT with 50 µM ZNS (right). WT is wild-type and MT is mutant Ca_V_3.1. **b** Comparison of Ca^2+^ current density at a holding potential of − 30 mV. No significant difference was observed between WT without ZNS and each group (WT with 50 µM ZNS, p = 0.588; MT without ZNS, p = 0.355; MT with 50 µM ZNS, p = 0.816)

Wild-type or mutant *CACNA1G* was transfected into HEK293T cells, and whole-cell recordings were made from GFP-positive cells. To isolate voltage-dependent Ca^2+^ currents, HEK293T cells were bathed with extracellular solution containing potassium channel blockers. No detectable current was observed in non-transfected HEK293T cells. In contrast, in HEK293T cells expressing the wild-type or mutant Ca_V_3.1 construct, step voltage changes following the preceding hyperpolarizing potential elicited rapidly-inactivating inward currents (Fig. 1a).

**Fig. 2:**
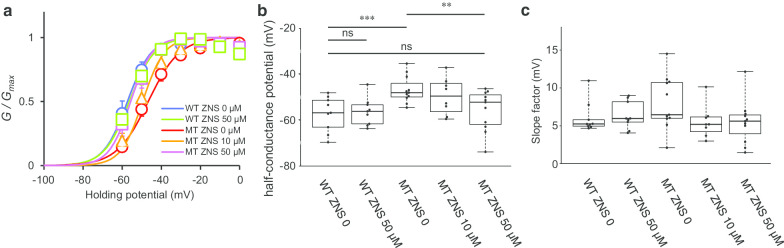
Detailed analysis of the effect of ZNS on mutant Ca_V_3.1. **a** Steady-state activation curves with ZNS. The normalized conductance-voltage curve was fitted to a Boltzmann equation. The activation curve of MT was shifted toward negative membrane potentials by ZNS in a concentration-dependent manner and approached the activation curve of WT. Data are presented as the mean ± S.E.M. (WT ZNS 0 [n = 9], WT ZNS 50 µM [n = 9], MT ZNS 0 [n = 11], MT ZNS 10 µM [n = 8], and MT ZNS 50 µM [n = 11]). **b** Comparison of the half-conductance potential. Data were obtained from the same cells shown in Fig. 1. There was a significant difference between WT and MT without ZNS (p = 0.002). In mutant Ca_V_3.1, ZNS significantly reduced the half-conductance of the activation potential (p = 0.006). No significant difference was observed between WT without ZNS and with 50 µM ZNS (p = 0.782) and between WT without ZNS and MT with 50 µM ZNS (p = 0.625). **p < 0.01, ***p < 0.005, ns: not significant. **c** Comparison of the slope factor of the activation curve. There was no significant difference between WT without ZNS and each group (WT with 50 µM ZNS, p = 0.808; MT without ZNS, p = 0.110; MT with 10 µM ZNS, p = 0.677; MT with 50 µM ZNS, p = 0.614)

To examine the pharmacological effects of ZNS on the Ca_V_3.1 currents, we made whole-cell recordings in the presence or absence of different concentrations of ZNS in the external solution and compared the Ca_V_3.1 currents. The current densities (absolute peak amplitude of the Ca_V_3.1 current divided by capacitances) did not decrease significantly in the presence of 50 µM ZNS (Fig. 1b). To examine changes in the voltage dependence of the mutant Ca_V_3.1, the estimated relative conductance was plotted against the depolarizing membrane potential steps and fitted by the Boltzmann equations (see “Methods”). As reported previously [1], in the external control solution, the half-conductance of the activation potential of mutant Ca_V_3.1 was significantly positive compared to that of wild-type Ca_V_3.1 (wild-type Ca_V_3.1, − 57.3 ± 7.6 mV [mean ± SD], mutant Ca_V_3.1, − 46.7 ± 5.9 mV without ZNS; p = 0.002). We found that ZNS shifted the half-conductance of the activation potential of mutant Ca_V_3.1 to more hyperpolarized potentials in a concentration-dependent manner. In mutant Ca_V_3.1, increasing the concentration of ZNS from 0 to 50 µM significantly reduced the half-conductance of the activation potential (p = 0.006) (Fig. 2a, b). Meanwhile, in wild-type Ca_V_3.1, the half-conductance of the activation potential with 50 µM ZNS was not statistically significant from that without ZNS (p = 0.782). Moreover, the difference between the half-conductance of the activation potential of wild-type Ca_V_3.1 without ZNS and that of mutant Ca_V_3.1 with 50 µM ZNS was not significant (p = 0.625). These data suggest that ZNS has the potential to shift mutant Ca_V_3.1 activation to more hyperpolarized potentials. The slope factor of the activation curve was not affected by the presence of the mutation and the concentration of ZNS, suggesting that the activation curve of the mutant Ca_V_3.1 shifted to negative membrane potentials in a parallel manner (Fig. 2c). In contrast to the activation, the steady-state inactivation was not affected by ZNS. The half-conductance of the inactivation potential of wild-type Ca_V_3.1 without ZNS [n = 7] was not significantly different from each group (wild-type with 50 µM ZNS [n = 11], p = 0.576; mutant without ZNS [n = 8], p = 0.704; mutant with 10 µM ZNS [n = 8], p = 0.803; mutant with 50 µM ZNS [n = 10], p = 0.992). The slope factor of the inactivation curve of wild-type Ca_V_3.1 without ZNS was also not significantly different from each group (wild-type with 50 µM ZNS, p = 0.460; mutant without ZNS, p = 0.912; mutant with 10 µM ZNS, p = 0.443; mutant with 50 µM ZNS, p = 0.180). These results suggest that ZNS can ameliorate the positive shift of the mutant Ca_V_3.1 activation curve without significantly influencing the absolute amplitude.

### Efonidipine did not affect the electrophysiological properties of mutant Ca_V_3.1

**Fig. 3:**
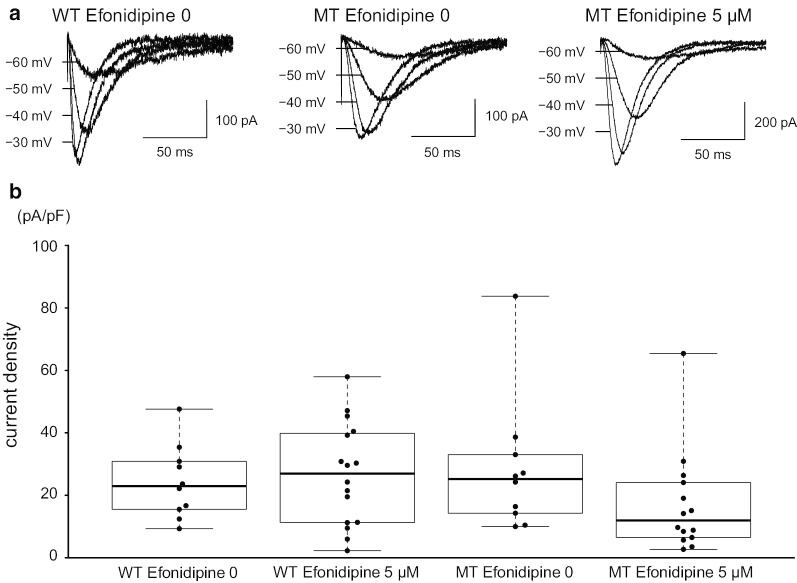
Effect of efonidipine on mutant Ca_V_3.1. **a** Representative traces of T-type VDCC currents recorded from HEK293T cells expressing WT without efonidipine (left), MT without efonidipine (middle), or MT with 5 µM efonidipine (right). **b** Comparison of the Ca^2+^ current density at a holding potential of − 40 mV. No significant difference was observed between WT without efonidipine and each group (WT with 5 µM efonidipine, p = 0.727; MT without efonidipine, p = 0.585; MT with 5 µM efonidipine, p = 0.312)

**Fig. 4:**
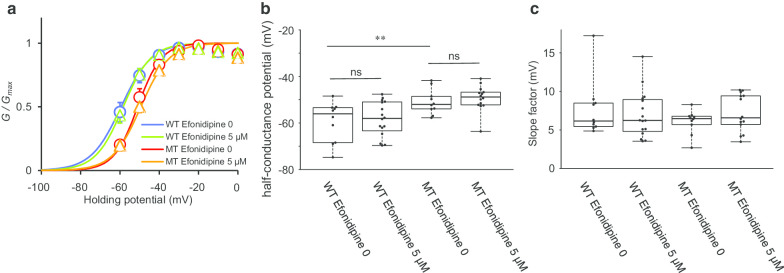
Detailed analysis of the effect of efonidipine on mutant Ca_V_3.1. **a** Steady-state activation curves with efonidipine. The activation curve of MT did not shift with the administration of efonidipine. Data are presented as the mean ± S.E.M. (WT efonidipine 0 [n = 10], WT efonidipine 5 µM [n = 16], MT efonidipine 0 [n = 10], and MT efonidipine 5 µM [n = 14]). **b** Comparison of the half-conductance of the activation potential. Data were obtained from the same cells shown in Fig. 3. There was a significant difference between WT and MT without efonidipine (p = 0.008). However, efonidipine did not affect the half-conductance of the activation potential of wild-type and the mutant Ca_V_3.1 significantly (p = 0.695, 0.567, respectively). **p < 0.01, ns: not significant. **c** Comparison of the slope factor of the activation curve. No significant difference was observed between WT without efonidipine and each group (WT with 5 µM efonidipine, p = 0.550; MT without efonidipine, p = 0.200; MT with 5 µM efonidipine, p = 0.695)

We showed the possibility of modulating the aberrant shift in the voltage dependence of the mutant Ca_V_3.1 by ZNS. Additionally, we examined whether other T-type VDCC blockers showed the same effect. We used efonidipine because it is available in Japan as an L- and T-type calcium channel blocker and was administered to our patient with SCA42. Whole-cell recordings were made in the presence and absence of efonidipine in the external solution, and Ca_V_3.1 currents were compared (Fig. 3a). Efonidipine insignificantly attenuated the Ca^2+^ current density mediated by the mutant Ca_V_3.1 (Fig. 3b). In contrast to ZNS, efonidipine did not affect the half-conductance of the activation potential of wild-type (p = 0.695) and mutant Ca_V_3.1 (p = 0.567) (Fig. 4a, b). The slope factor of the activation curve was also not affected by the presence of the mutation and the concentration of efonidipine (Fig. 4c). These data indicate that efonidipine did not ameliorate the abnormal shift in the activation curve of the mutant Ca_V_3.1.

## Discussion

We found that ZNS modulated the abnormal voltage dependence of the mutant Ca_V_3.1. Several studies have demonstrated that ZNS modulates the voltage dependence of Ca_V_3.1. ZNS decreases the peak current of T-type calcium channels and changes the voltage dependence of T-type VDCC [9, 10]. ZNS induced the hyperpolarizing shift of the voltage dependence of T-type calcium channel inactivation in cultured neuroblastoma cells [9]. However, there was no description of the channel activation, and, unlike our study, the currents of all subtypes of T-type VDCC in cultured cells were measured using endogenous channel expression rather than Ca_V_3.1 overexpression. Matar et al. showed that the activation potential of Ca_V_3.2 was significantly negatively shifted with 100 μM ZNS, but the half-inactivation potential of Ca_V_3.2 was not shifted. As for Ca_V_3.1, only a slight reduction in the inactivation-tau was shown, while the activation shift and inactivation curve were not shown [10]. In the present study, we showed that low-dose ZNS shifted the activation curve of the mutant Ca_V_3.1 to negative potentials. According to previous reports, ZNS may change the voltage dependence of VDCC activity; our results add to these findings. Because this study was performed using a physiologically relevant concentration of ZNS calculated based on reports that the blood and brain concentrations are approximately equivalent, our results suggest that ZNS shifted the abnormal voltage dependence of the mutant Ca_V_3.1 in the patient with SCA42. Calhoun et al. demonstrated that the level of *Cacna1g* expression was associated with the severity of epilepsy in a mouse model with a mutation of the voltage-gated sodium channel *Scn2a* [13, 14]. As ZNS is also a sodium channel blocker [8], the effects of sodium channel blockade may have an indirect influence on tremor in patients with SCA42.

Neurons are known to fire in different patterns in response to membrane depolarization, which occurs in single-spike mode and burst mode. The single-spike mode is elicited by depolarization from around the resting membrane potential, whereas the burst mode is elicited by depolarization from a deeper potential (less than − 70 mV) [15]. T-type calcium channel activation facilitates initiation of the burst firing [15], and T-type calcium channel blockers inhibit the initial increase in burst firing and shorten the burst duration [16, 17]. In PD, motor symptoms such as bradykinesia are reportedly involved in burst firing elicited by T-type calcium channels of the subthalamic nucleus [18, 19]. It has also been reported that bradykinesia in PD model rats was improved by the administration of T-type calcium channel inhibitors such as nickel, mibefradil, and NNC 55-0396 or by altering the firing pattern by STN-DBS. These data support the relationship between burst firing induced by T-type calcium channels and the pathophysiology of PD. Similarly, the generation of tremors is thought to be related to T-type calcium channels in neural structures that are widely distributed in the brain, such as in the inferior olive nucleus, Purkinje cells, deep cerebellar nucleus, thalamus, basal ganglia, and spinal cord [20]. Analysis of harmaline-induced tremors in mice showed that the Ca^2+^ current is an important factor in tremor generation with burst firing induced from Ca_V_3.1 of the inferior olive nucleus propagating to the deep cerebellar nucleus and synchronizing adjacent neurons in sequence [11]. As previously reported, inhibition of the Ca^2+^ current contributes to the improvement of tremors [21]. These lines of evidence suggest that changes in the firing pattern of neurons, which are caused by the mutation of Ca_V_3.1, may generate tremors in SCA42 patients [1].

Before treatment with a low-dose of ZNS, we attempted to treat intractable tremors in the patient with SCA42 using efonidipine, but no therapeutic effect was observed. Although we cannot rule out the possibility of poor efonidipine permeability through the blood–brain barrier, the present study demonstrated that effective doses of efonidipine as an antihypertensive agent did not affect the voltage dependence of the mutant Ca_V_3.1. These results suggest that ZNS has unique properties compared to other T-type calcium channel blockers, in terms of modulating the voltage dependence of the mutant Ca_V_3.1. There are several reports of the neuroprotective effects of ZNS in PD. ZNS has also been suggested to delay disease progression [22–24]. In our study, low-dose ZNS was effective for treating intractable tremors in the patient with SCA42. Studies are needed to determine whether continuous administration of ZNS starting in the early stages of SCA42 is effective against symptoms other than tremor and useful in delaying disease progression.

In conclusion, the membrane potential at half activation of the mutant Ca_V_3.1 was shifted positively and approached that of the wild-type in a concentration-dependent manner following ZNS exposure. Although ZNS is well-known as a T-type calcium channel blocker, our experiments revealed that it also acts as a modulator to ameliorate the voltage dependence of Ca_V_3.1 mutation, causing SCA42.

## Data Availability

Not applicable.

## Abbreviations

PD: Parkinson’s disease
SCA: Spinocerebellar ataxia
VDCC: Voltage-dependent calcium channel
ZNS: Zonisamide
